# Three-Dimensional Surface Topography for Entropy-Based Comparison of Sagittal Postural Classifications in Children

**DOI:** 10.3390/s26175408

**Published:** 2026-08-27

**Authors:** Jacek Wilczyński, Małgorzata Gawlik, Katarzyna Bieniek, Paulina Szumilas, Natalia Habik-Tatarowska, Kamil Markowski, Wiktoria Świetlak, Agata Michalska

**Affiliations:** Laboratory of Posturology, Collegium Medicum, Jan Kochanowski University of Kielce, 25-317 Kielce, Poland; gawlik.malgosia@wp.pl (M.G.); katarzyna.bieniek@onet.com.pl (K.B.); palulinal@gmail.com (P.S.); habiknatalia@gmail.com (N.H.-T.); kamil.markowski7@gmail.com (K.M.); wiktoria.sw@op.pl (W.Ś.); agata.michalska.2@ujk.edu.pl (A.M.)

**Keywords:** rasterstereography, 3D surface topography, sensor-based measurement, sagittal spinal alignment, thoracic kyphosis, lumbar lordosis, sagittal posture classification, Shannon entropy, classification granularity

## Abstract

**Highlights:**

**What is the main finding?**

- Applying the operationalised five-category classification and the nine-type Wilczyński typology to the same sensor-derived continuous thoracic kyphosis and lumbar lordosis measurements produced substantially different categorical distributions. The nine-type typology yielded a more even and dispersed distribution of sagittal postural profiles, as reflected by higher normalised Shannon entropy.

**What is the implication of the main finding?**

- Categorical representations of sagittal posture depend on the thresholds and category architecture applied to the underlying sensor-derived continuous measurements. Combining sensor-based three-dimensional rasterstereography with transparent classification criteria and the retention of continuous data supports methodological standardisation and reproducibility, while Shannon entropy should be interpreted as a measure of distributional structure rather than clinical validity.

**Abstract:**

Sensor-based three-dimensional surface topography provides non-invasive quantitative measurements of sagittal spinal alignment, but categorical posture profiles also depend on the thresholds and category structure used to transform continuous measurements. This cross-sectional methodological study compared an operationalised five-category classification with the nine-type Wilczyński typology using the same sensor-derived thoracic kyphosis and lumbar lordosis measurements obtained with the DIERS Formetric III 4D rasterstereographic system. A total of 952 children aged 10–12 years (478 girls, 474 boys) were assessed. Category distributions were summarised descriptively, cross-classified in a 9 × 5 contingency table, and evaluated using Cramér’s *V* and Shannon entropy. The five-category classification concentrated 94.1% of participants in two categories, whereas the nine-type typology produced a more dispersed and even distribution. Normalised Shannon entropy was higher for the nine-type typology (0.85 vs. 0.57), and Cramér’s *V* was 0.55. Because both systems were deterministic transformations of the same sensor-derived measurements, these statistics were interpreted descriptively. The findings show that classification thresholds and category architecture substantially influence categorical representations derived from identical sensor-based measurements. Greater granularity and higher entropy indicate distributional differentiation, not superior clinical validity; independent external validation is required to establish clinical relevance.

## 1. Introduction

Accurate assessment of sagittal spinal alignment is an important component of musculoskeletal evaluation in children and adolescents. Thoracic kyphosis and lumbar lordosis are fundamental components of the sagittal spinal profile and undergo substantial changes during growth and maturation [1,2,3]. Their magnitude shows considerable interindividual variability. Sagittal posture has also been investigated in relation to age, body composition, weight status, and sports participation [4,5,6,7], with associations varying across study designs and outcome measures. For this reason, objective and reproducible assessment of sagittal spinal alignment is particularly important in paediatric populations.

The development of three-dimensional (3D) surface topography has enabled the non-invasive, radiation-free acquisition of quantitative information on back-surface geometry. Rasterstereography was introduced as a photogrammetric method for three-dimensional measurement of body surfaces [8], while subsequent work established surface-curvature analysis for quantitative assessment of back shape [9]. Drerup and Hierholzer subsequently developed methods for the objective identification of anatomical landmarks on the body surface [10], automatic localisation of anatomical landmarks and construction of a body-fixed coordinate system [11], and three-dimensional reconstruction of spinal shape from rasterstereographic back-surface data [12]. Precision measurement of thoracic kyphosis and lumbar lordosis using rasterstereography was further described by Hierholzer and Drerup [13]. These studies established the geometric and photogrammetric foundations on which subsequent rasterstereographic systems were developed.

Later research evaluated the measurement properties and applicability of rasterstereography. Guidetti et al. demonstrated high intra- and interday reliability for most parameters measured using the Formetric 4D system [14]. A systematic review by Su et al. subsequently reported generally satisfactory reliability and validity of surface-topography measurements, including Formetric-derived parameters, while also emphasising variability among individual measures and study protocols [15]. Betsch et al. evaluated the reliability and validity of 4D rasterstereography under dynamic experimental conditions [16]. Knott et al. compared DIERS Formetric surface-topography measurements directly with conventional radiographic measurements in paediatric patients with spinal deformity [17], whereas Furian et al. demonstrated the feasibility of rasterstereographic assessment of spinal posture and pelvic position in a large sample of elementary-school children [18]. Contemporary reviews have further summarised the technologies, methodological principles, and clinical utility of non-invasive back-surface topography [19]. Accordingly, the DIERS Formetric III 4D system used in the present study provides a sensor-based optical measurement framework in which continuous quantitative angular parameters can subsequently serve as inputs to predefined posture classification systems.

Transforming sensor-derived continuous measurements of thoracic kyphosis and lumbar lordosis into posture categories requires the application of specific decision thresholds. Traditional clinical and morphotype-based approaches distinguish a limited number of sagittal postural patterns on the basis of thoracic and lumbar curvature characteristics [20,21,22]. For the purposes of the present study, these concepts were operationalised a priori into a five-category framework comprising normal posture, round back, concave back, round-concave back, and flat back. The resulting category assignment depends on the reference ranges, thresholds, and category structure applied [20,21,22]. The threshold values used in the present analysis represent an operational definition and should not be regarded as a universal paediatric diagnostic standard. An alternative approach is the nine-type Wilczyński typology, based on a two-dimensional 3 × 3 combinatorial classification [23]. In this framework, both thoracic kyphosis and lumbar lordosis are classified as reduced, within the adopted reference interval, or increased, and the combination of these three levels for both curvatures produces nine mutually exclusive sagittal postural profiles [23]. This system extends classifications based on broader posture categories by systematically incorporating the full set of combinations of thoracic kyphosis and lumbar lordosis, thereby allowing a more detailed representation of the co-occurrence of configurations of both curvatures [23]. However, greater detail is a property of the classification structure itself and does not, in itself, imply greater biological, diagnostic, or clinical validity.

Because the nine-type typology was previously proposed by the corresponding author [23], the present study was not designed as an independent validation of this system or as an assessment of its clinical superiority, but rather as a methodological comparison of two predefined classification structures applied to the same sensor-derived continuous measurements. A key methodological issue is therefore that different classification systems may transform the same sensor-derived thoracic kyphosis and lumbar lordosis values into different posture categories [20,21,22,23]. A child may be assigned to a different profile not because the sensor-derived measurement itself has changed, but because a different decision threshold, number of categories, or classification algorithm has been applied. This is relevant both to the interpretation of the prevalence of particular posture types and to comparisons of results across studies. Some of the differences observed between populations may therefore reflect the way in which sensor-derived continuous data are categorised rather than true differences in the underlying continuous parameters. Most previous studies have focused on the values and prevalence of different spinal curvature configurations and on their associations with developmental, anthropometric, and environmental factors [1,2,3,4,5,6,7,20,21,22]. Considerably less attention has been paid to how different classification systems, when applied to the same sensor-derived measurements of thoracic kyphosis and lumbar lordosis, influence the allocation of participants across individual posture categories. In particular, it remains important to determine the extent to which the number of categories and the adopted threshold values affect the distribution of participants across postural profiles and the level of classification granularity obtained.

Shannon entropy provides a quantitative measure of the distribution of observations across categories [24]. In the present application, it reflects the degree of evenness with which participants are distributed across predefined postural profiles. Because the maximum possible entropy depends on the number of available categories, comparison of systems containing different numbers of categories also requires the use of normalised entropy, which expresses the observed value relative to the theoretical maximum for a given system [24]. However, both absolute and normalised entropy remain measures describing distributional structure and do not constitute criteria of clinical validity, diagnostic accuracy, or superiority of a given system. A higher entropy value should therefore be interpreted as reflecting a more even and dispersed distribution of observations across the available categories rather than as evidence of greater clinical usefulness.

Nevertheless, comparing alternative classification structures applied to identical sensor-derived measurements may provide important methodological information. In particular, it enables assessment of the extent to which the observed prevalence of individual profiles depends on the structure of the classification system. This is relevant to transparent reporting of sensor-based measurement procedures and classification criteria and to maintaining comparability of results across studies and repeated assessments. By contrast, determining whether a more detailed classification provides additional clinical, functional, prognostic, or monitoring value requires independent validation using appropriate external reference outcomes.

To the best of our knowledge, the distributional properties of alternative sagittal posture classification systems, assessed using Shannon entropy and based on the same sensor-derived measurements obtained by three-dimensional surface topography, have not previously been analysed in a large paediatric population. Therefore, the aim of the present study was to compare the distribution of sagittal postural profiles obtained using an operationalised five-category classification and the nine-type Wilczyński typology, both applied to the same sensor-derived thoracic kyphosis and lumbar lordosis measurements obtained by 3D surface topography. Specifically, we evaluated participant allocation across categories, Shannon entropy and normalised entropy, and the correspondence between the two classification structures. We hypothesised that the nine-type classification would result in a more even distribution of participants across the available posture categories than the five-category system, as reflected primarily by higher normalised Shannon entropy. Absolute Shannon entropy was additionally reported as a descriptive measure of category-distribution structure. This hypothesis concerns only the distributional properties of the two classification systems and does not imply greater clinical validity, diagnostic accuracy, or superiority of either system.

## 2. Materials and Methods

### 2.1. Characteristics of the Study Group

This cross-sectional study included 952 children aged 10–12 years attending primary schools in the Świętokrzyskie region of Poland. The study group comprised 478 girls (50.2%) and 474 boys (49.8%). Data were collected during the 2019–2020 school year. Participants were recruited using non-probability convenience sampling from primary schools that agreed to participate in the project. Accordingly, the study sample should be regarded as a regional convenience sample rather than as a population-representative cohort. Parents or legal guardians received written information describing the aims and procedures of the study and provided written informed consent before examination. Complete recruitment records documenting the total number of schools approached, the number of schools that declined participation, and the total number of children initially invited were not available. Consequently, school-level and individual-level participation rates could not be reliably calculated, and the possibility of selection bias related to school or family participation cannot be excluded. Children were eligible for inclusion if they were 10–12 years of age and had no known diagnosis of a neurological, orthopaedic, congenital, neuromuscular, genetic, endocrine, or other disorder considered likely to substantially affect spinal alignment or habitual body posture. Exclusion criteria comprised certified physical or intellectual disability, a previously diagnosed orthopaedic disorder affecting posture or spinal alignment, and the absence of written informed consent from a parent or legal guardian. No additional standardised clinical or radiographic scoliosis-screening protocol was systematically applied to the entire study cohort. Radiographs were not obtained for research purposes because the study was based on non-invasive surface-topography measurements and did not involve exposure to ionising radiation. Eligibility with respect to pre-existing spinal disorders was therefore determined from the available medical and health information rather than by systematic radiographic verification of the absence of structural scoliosis. Consequently, the possibility that some children with previously unrecognised spinal deformity were included cannot be completely excluded.

The age range of 10–12 years was selected because it represents a developmental period characterised by substantial changes in body proportions and sagittal spinal alignment associated with growth and the transition toward adolescence [1,2,3]. Restricting the cohort to a relatively narrow chronological age range reduced broad age-related heterogeneity; however, chronological age is not equivalent to biological maturity. Tanner stage, skeletal age, peak height velocity, maturity offset, age at peak height velocity, and other direct or surrogate indicators of pubertal maturation were not assessed. Consequently, maturational heterogeneity within the cohort could not be quantified or statistically controlled. This limitation is relevant because sagittal spinal alignment changes during childhood and pubertal growth [1,2,3]. The present cohort partially overlapped with the sample used in the earlier study in which the nine-type Wilczyński classification was originally described [23]. Specifically, all 303 participants from the original cohort were included in the present dataset of 952 children, corresponding to 31.8% of the current sample. The remaining 649 participants constituted additional observations that were not included in the earlier study. This overlap is explicitly acknowledged because it affects the interpretation of the present findings. Accordingly, the current study was not designed as an independent external validation of the nine-type classification and should not be interpreted as such. Rather, its purpose was to compare the distributional characteristics of two predefined deterministic sagittal posture classification systems when applied to the same two continuous sensor-derived variables—thoracic kyphosis angle and lumbar lordosis angle—in an enlarged dataset. The analysis therefore concerns differences in classification granularity and distributional structure rather than the diagnostic superiority, clinical validity, or external validation of either classification system.

The study was conducted in accordance with the Declaration of Helsinki and approved by the Bioethics Committee of Jan Kochanowski University in Kielce, Poland (Approval No. 37/2018). The principal demographic characteristics and sagittal spinal alignment parameters of the study group are presented in Table 1. The mean age of the participants was 10.91 ± 0.79 years. The mean thoracic kyphosis angle was 41.12 ± 11.53°, whereas the mean lumbar lordosis angle was 40.49 ± 10.12°. These continuous variables are reported because they constituted the primary sensor-derived measurements obtained using surface topography and were subsequently used to assign participants to the respective posture categories. The reported means and standard deviations are descriptive characteristics of the study population only and were not used to establish reference ranges, classification cut-offs, or category thresholds. In particular, the standard deviations reflect interindividual variability within the study sample and should not be interpreted as reference intervals or as criteria defining normal or abnormal sagittal spinal alignment. Posture classification was performed individually for each participant on the basis of the measured combination of thoracic kyphosis and lumbar lordosis angles and the predefined decision rules of the respective classification system. Category assignment was therefore based on predetermined classification criteria rather than on sample-derived means, standard deviations, percentiles, or other distribution-dependent cut-offs. As shown in Table 1, categorical variables are presented as *n* (%), whereas continuous variables are reported as mean ± standard deviation. Because the predefined objective of the present study was to compare the distributional properties of two posture classification systems applied to the same underlying sensor-derived continuous measurements, analyses of sex-related differences, anthropometric characteristics, pubertal status, and other potential biological or environmental determinants of sagittal spinal alignment were not included in the primary analysis. Although these factors may independently influence sagittal posture, their analysis would address a different research question from the methodological comparison undertaken in the present study.

### 2.2. Research Tools and Body Posture Assessment

Body posture was assessed using the DIERS Formetric III 4D system (DIERS International GmbH, Schlangenbad, Germany), a non-invasive sensor-based optical rasterstereographic system for three-dimensional assessment of back-surface geometry and model-based reconstruction of spinal and pelvic alignment without the use of ionising radiation [12,14,19]. The photogrammetric foundations of rasterstereography were established by Frobin and Hierholzer [8,9] and were subsequently developed by Drerup and Hierholzer for objective identification of anatomical landmarks, construction of a body-fixed coordinate system, and three-dimensional reconstruction of spinal shape [10,11,12]. Precision measurement of kyphosis and lordosis using rasterstereography was subsequently described by Hierholzer and Drerup [13], while the methodological principles and clinical applications of video rasterstereography were also discussed by Harzmann [25].

Rasterstereography projects a structured pattern of parallel light lines onto the participant’s back and records their deformation using an optical camera. Photogrammetric analysis of the distorted raster enables reconstruction of back-surface geometry, localisation of anatomical landmarks, and model-based estimation of spinal shape and surface rotation [8,9,10,11,12]. The sensor-based acquisition and reconstruction process therefore provides quantitative information on back-surface geometry and model-based estimates of spinal alignment without exposure to ionising radiation [17,19].

The DIERS Formetric III 4D system was operated and calibrated according to the manufacturer’s recommendations. All measurements were performed under standardised conditions by investigators experienced in the DIERS acquisition protocol. Participants were examined barefoot, with the back exposed, in a habitual, relaxed bipedal standing position. They were instructed to stand naturally, distribute body weight between both lower limbs, keep the upper limbs relaxed alongside the trunk, maintain the head in a neutral position, and avoid intentional postural correction. Comparable standing protocols have been used in studies evaluating Formetric reliability and paediatric spinal posture [14,18]. Each examination was performed using the 4D Average acquisition mode, which is based on serial image acquisition and averaging to reduce the influence of short-term postural fluctuations and minor involuntary movements on the resulting values [14,19]. The exact number of frames contributing to the averaged measurement could not be retrospectively verified from the archived records and is therefore not reported.

Following sensor acquisition, DICAM 3 software (version 3.0; DIERS International GmbH, Schlangenbad, Germany) automatically reconstructed the back surface and calculated spinal and pelvic parameters. The present analysis used only two continuous sensor-derived sagittal-plane variables: the thoracic kyphosis angle ICT–ITL (max) and the lumbar lordosis angle ITL–ILS (max) [13,14]. Other Formetric parameters, including frontal-plane deviation, surface rotation, trunk alignment, and pelvic position, were not used for posture classification [14,19]. The sagittal angles analysed in the present study were derived from surface topography and should not be regarded as directly interchangeable with radiographic measurements [15,17].

The measurement properties of rasterstereography have been evaluated in primary methodological and validation studies. Drerup and Hierholzer reported root-mean-square deviations of approximately 4 mm between radiographic and rasterstereographically reconstructed spinal midlines and approximately 3° between vertebral rotation and surface rotation in patients with scoliosis [12]. These values are specific to the parameters and conditions investigated and should not be interpreted as universal measurement errors for all DIERS-derived variables. Guidetti et al. reported excellent intra- and interday reliability for most Formetric 4D parameters, although reliability varied among individual variables [14]. Betsch et al. demonstrated satisfactory reliability and validity of 4D rasterstereography under dynamic experimental conditions; however, their findings should be regarded as supporting the technological principles of 4D rasterstereography rather than as direct validation of the static sagittal measurements used in the present study [16]. In paediatric populations, Knott et al. compared DIERS Formetric measurements with conventional radiography in patients aged 8–18 years and reported strong reproducibility, although agreement with radiographic measurements varied according to the spinal parameter and was stronger for thoracic than for lumbar measurements [17]. Su et al. likewise reported satisfactory reliability and validity for many surface-topography parameters while emphasising parameter-specific differences in measurement performance [15]. Furian et al. demonstrated the feasibility of rasterstereographic assessment in 345 school-aged children, including quantitative evaluation of thoracic kyphosis and lumbar lordosis [18]. Additional studies have compared surface-topography or rasterstereographic assessments with radiographic or orthopaedic reference methods [26,27]. Collectively, these findings support the use of rasterstereography as a quantitative measurement technique while indicating that reliability and validity should be interpreted in a parameter- and protocol-specific manner [14,15,16,17,18,19,26,27].

Posture classification was performed only after acquisition of the continuous sensor-derived thoracic kyphosis and lumbar lordosis measurements. Predefined numerical decision rules were subsequently applied to these two variables. Category assignment was therefore deterministic: once the thoracic kyphosis (*K*) and lumbar lordosis (*L*) values had been obtained, examiner judgement did not influence allocation to a posture category. Thus, uncertainty associated with the sensor-based measurement process and threshold-dependent classification uncertainty represent distinct methodological issues.

Two categorical frameworks were applied to the same continuous sensor-derived *K* and *L* measurements. The first was an operationalised five-category classification comprising normal posture, round back, concave back, round-concave back, and flat back. Its conceptual basis was consistent with conventional descriptions of sagittal postural patterns [20] and with paediatric studies classifying sagittal morphotypes according to thoracic and lumbar curvature characteristics [21,22]. Because these sources do not provide the exact numerical decision algorithm used in the present study, the thresholds were treated as an a priori operationalisation rather than as diagnostic criteria directly derived from the literature [20,21,22]. Round back was defined as *K* > 40° and *L* ≤ 50°; round-concave back as *K* > 40° and *L* > 50°; concave back as *K* ≤ 40° and *L* > 50°; and flat back as *K* < 20° and *L* < 30°. Normal posture comprised all remaining observations. These criteria were applied identically to all participants and should not be interpreted as universally validated paediatric diagnostic thresholds.

The second framework was the nine-type Wilczyński typology [23], based on a two-dimensional combinatorial 3 × 3 classification. Thoracic kyphosis was classified as reduced (*K* < 42°), within the adopted reference interval (42° ≤ *K* ≤ 55°), or increased (*K* > 55°), whereas lumbar lordosis was classified as reduced (*L* < 33°), within the adopted reference interval (33° ≤ *L* ≤ 47°), or increased (*L* > 47°) [23]. Combining these categories generated nine mutually exclusive sagittal postural profiles. The terms reduced, within the adopted reference interval, and increased refer only to the numerical boundaries adopted within this classification framework and should not be interpreted as independent clinical diagnoses or universally applicable paediatric normative values [1,2,3,4,23].

**Figure 1:**
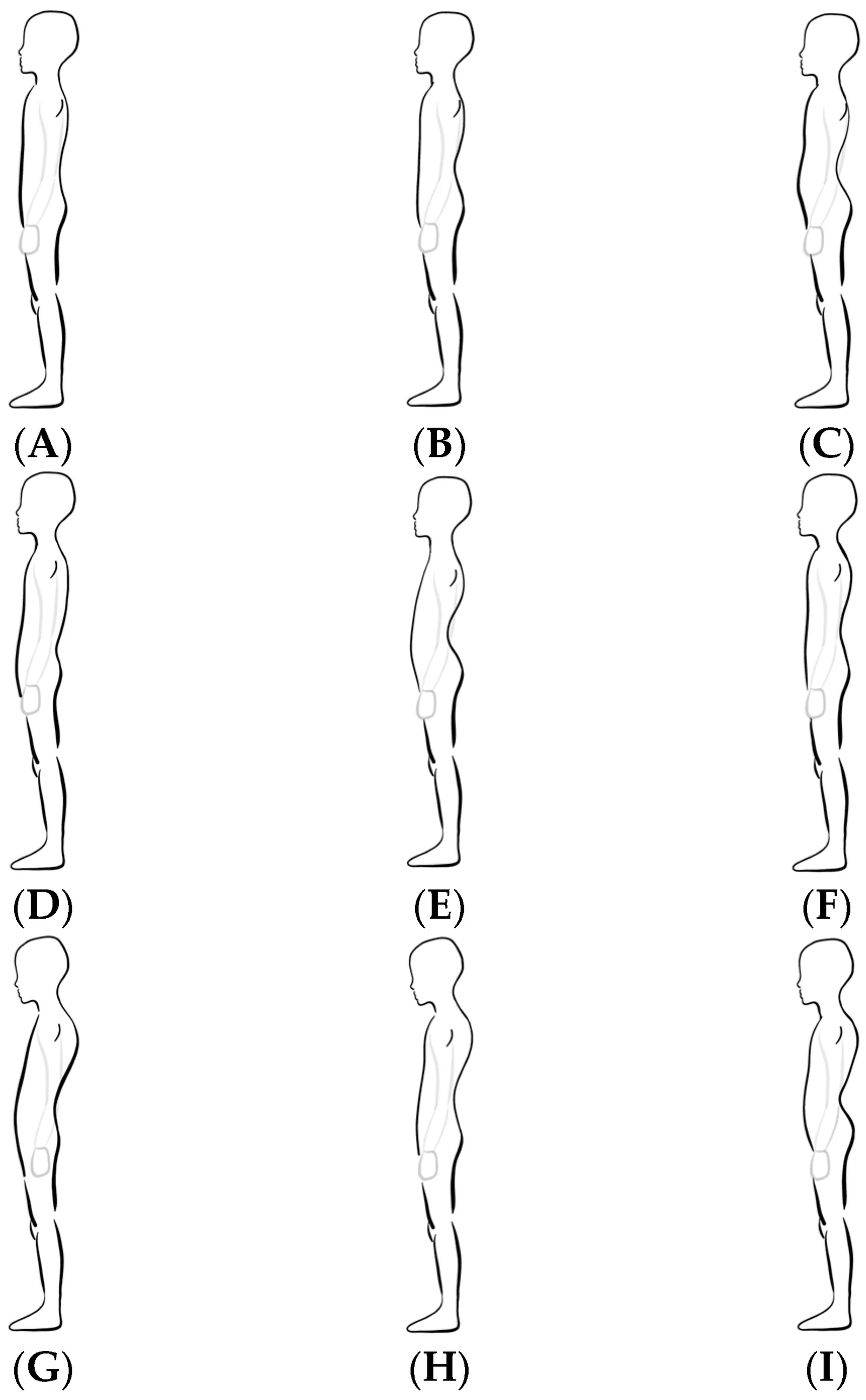
Author-created schematic representation of the nine-type Wilczyński sagittal posture classification based on thoracic kyphosis and lumbar lordosis angles. (**A**) Reduced thoracic kyphosis and reduced lumbar lor-dosis (*K* < 42°; *L* < 33°). (**B**) Reduced thoracic kyphosis and lumbar lordosis within the adopted reference interval (*K* < 42°; 33° ≤ *L* ≤ 47°). (**C**) Reduced thoracic kyphosis and increased lumbar lordosis (*K* < 42°; *L* > 47°). (**D**) Thoracic kyphosis within the adopted reference interval and reduced lumbar lordosis (42° ≤ *K* ≤ 55°; *L* < 33°). (**E**) Thoracic kyphosis within the adopted refer-ence interval and lumbar lordosis within the adopt-ed reference interval (42° ≤ *K* ≤ 55°; 33° ≤ *L* ≤ 47°). (**F**) Thoracic kyphosis within the adopted refer-ence interval and in-creased lumbar lordosis (42° ≤ *K* ≤ 55°; *L* > 47°). (**G**) Increased thoracic kyphosis and reduced lumbar lordosis (*K* > 55°; *L* < 33°). (**H**) Increased thoracic kyphosis and lumbar lor-dosis within the adopted reference interval (*K* > 55°; 33° ≤ *L* ≤ 47°). (**I**) Increased thoracic ky-phosis and increased lum-bar lordosis (*K* > 55°; *L* > 47°).

Figure 1 was created by the authors specifically for the present manuscript to illustrate the 3 × 3 structure of the nine-type Wilczyński typology. It was not reproduced or adapted from a previously published figure, does not introduce additional measurement or classification criteria, and was not used in data acquisition, processing, or statistical analysis.

Because rasterstereographic measurements are subject to parameter- and protocol-specific variability [14,15,16,17], fixed classification cut-offs imply that values located close to a decision boundary may be assigned to an adjacent category following small measurement variations. This issue is particularly relevant when relatively narrow angular intervals are used and was considered when interpreting the categorical findings.

### 2.3. Statistical Analysis

The nine-type Wilczyński typology was implemented as a two-dimensional 3 × 3 combinatorial classification based on predefined reference intervals for thoracic kyphosis and lumbar lordosis [23]. Each sagittal curvature parameter was classified as reduced, within the adopted reference interval, or increased, and the combination of these three levels for both variables yielded nine mutually exclusive sagittal postural profiles. These terms refer exclusively to the predefined boundaries of the classification system and were not treated as universal paediatric normative or diagnostic categories. Category distributions for both classification systems were summarised using frequencies and percentages. Cross-classification of the nine-type Wilczyński typology and the operationalised five-category classification was presented in a 9 × 5 contingency table. Because both systems were deterministic transformations of the same two continuous variables- thoracic kyphosis and lumbar lordosis- the resulting table contained structural constraints and was not treated as a contingency table arising from two statistically independent classification procedures. For descriptive completeness, Pearson’s chi-square statistic was calculated as a measure of departure from statistical independence within the cross-classification table. Cramér’s *V* was calculated as a descriptive measure of the magnitude of cross-classification association using the following formula: *V* = √[χ^2^/(*N* × min(*r* − 1, *c* − 1))], where χ^2^ denotes the Pearson chi-square statistic, *N* is the total sample size, and *r* and *c* denote the numbers of rows and columns, respectively. Neither statistic was interpreted as evidence of independent agreement, predictive validity, or validation of one classification system by the other. Inspection of the expected cell counts demonstrated substantial sparsity.

Of the 45 cells, 24 (53.3%) had expected counts below 5 and 14 (31.1%) had expected counts below 1. In addition, 28 of the 45 possible category combinations were structurally impossible under the predefined deterministic decision rules. These structural zeros resulted from the mathematical relationship between the two classification algorithms rather than from low sampling frequency. Consequently, the assumptions underlying the conventional asymptotic Pearson chi-square test were not satisfied, and its asymptotic *p*-value was not used for confirmatory statistical inference. A fixed-margin Monte Carlo simulation with 100,000 replicates was additionally performed as a sensitivity analysis using the simulate.p.value option of the chisq.test function in R. Because this procedure preserves the marginal totals but does not impose the structural-zero constraints generated by the deterministic classification rules, simulated tables could contain category combinations that were impossible under the actual classification scheme. The resulting Monte Carlo *p*-value was therefore interpreted cautiously and was not regarded as evidence of an association between two independently defined classification systems. Rare categories were not collapsed because doing so would have altered the predefined structure and conceptual meaning of the classification systems under comparison.

Shannon entropy was used as a descriptive measure of the distributional evenness of participants across the available posture categories [24] and was calculated as *H* = −Σ*p_i_* ln(*p_i_*), where *p_i_* denotes the proportion of participants assigned to category *i*, and ln denotes the natural logarithm. Because the maximum attainable entropy depends on the number of available categories, maximum entropy was calculated as: *Hmax* = ln(*k*), where *k* denotes the total number of categories within a classification system. Normalised entropy was calculated as *H*/*Hmax* and is referred to hereafter as the normalised entropy index. This normalisation was particularly important because the two classifications contained different numbers of categories (five and nine, respectively). Higher values of absolute or normalised entropy indicate a more even and dispersed allocation of observations across the available categories. However, entropy was treated solely as a descriptive measure of distributional structure and was not interpreted as evidence of superior clinical validity, diagnostic accuracy, measurement precision, prognostic value, or clinical utility. All statistical calculations reported in the revised manuscript were independently verified using reproducible code written in R version 4.6.1 (R Foundation for Statistical Computing, Vienna, Austria). Only functions available in the base R stats package were used. For reproducibility of the Monte Carlo sensitivity analysis, the random-number generator seed was set to 20260811 before simulation.

## 3. Results

### 3.1. Distribution of Sagittal Posture Profiles According to the Five-Category Classification

The operationalised five-category classification showed a markedly uneven distribution of participants across sagittal postural profiles. Most children were classified as having round back or normal posture, whereas concave back, round-concave back, and flat back occurred much less frequently (Table 2). Round back was the most common category, followed by normal posture, while the remaining three categories accounted for only a small proportion of the study population.

### 3.2. Distribution of Sagittal Posture Profiles According to the Nine-Type Wilczyński Typology

The application of the nine-type Wilczyński typology revealed a more heterogeneous distribution of sagittal postural profiles within the study population. The largest groups comprised children with reduced thoracic kyphosis and lumbar lordosis within the adopted reference interval, as well as children with both curvatures within the adopted reference intervals. Compared with the five-category classification, the nine-type typology allocated participants across a broader range of posture categories, resulting in a more dispersed pattern of sagittal spinal configurations (Table 3).

### 3.3. Comparison of the Two Classification Systems

Cross-classification analysis demonstrated partial correspondence between the five-category classification and the nine-type Wilczyński typology. However, individual categories of the five-category system encompassed several distinct combinations of thoracic kyphosis and lumbar lordosis when examined within the nine-type framework. This was particularly evident among children classified as having round back, who were distributed across multiple sagittal configurations in the nine-type typology. The cross-classification of sagittal postural profiles between the two systems is presented in Table 4. Overall, the nine-type typology produced a more dispersed categorical representation of sagittal postural profiles than the five-category classification (Table 4).

The Pearson chi-square statistic calculated from the complete 9 × 5 contingency table was χ^2^ = 1142.80, with Cramér’s *V* = 0.55. Because both classification systems were deterministic transformations of the same two continuous measurements, Cramér’s *V* was interpreted solely as a descriptive measure of cross-classification association. Twenty-four of the 45 expected cell counts were below 5, including 14 below 1. In addition, 28 of the 45 possible category combinations were structurally impossible under the predefined classification rules. Consequently, the assumptions underlying the conventional asymptotic Pearson chi-square test were not satisfied. A fixed-margin Monte Carlo simulation with 100,000 replicates yielded *p* < 0.001. However, because the simulation did not incorporate the structural-zero constraints imposed by the deterministic classification rules, this result was treated only as a sensitivity analysis. It should therefore not be interpreted as evidence of an association between two independently defined classification systems (Table 5).

### 3.4. Entropy Analysis of the Two Classification Systems

Entropy analysis demonstrated differences in the distributional structure of posture categories between the two classification systems. Shannon entropy was higher for the nine-type Wilczyński typology than for the five-category classification (*H* = 1.87 vs. 0.91). The corresponding maximum entropy values were 2.20 and 1.61, respectively. Normalised entropy (*H*/*Hmax*) was also higher for the nine-type typology (0.85 vs. 0.57) (Table 6). These findings indicate a more even and dispersed allocation of participants across the available categories in the nine-type classification, whereas the five-category system showed greater concentration within a smaller number of categories.

## 4. Discussion

The present study compared the categorical distributions generated when two predefined sagittal posture classification frameworks were applied to the same sensor-derived continuous measurements of thoracic kyphosis and lumbar lordosis obtained using three-dimensional surface topography. The principal finding was that the categorical representation of sagittal posture depended substantially on the threshold structure and category architecture applied after sensor acquisition. The operationalised five-category classification concentrated 94.1% of participants in only two categories—round back and normal posture—whereas the nine-type 3 × 3 Wilczyński typology distributed participants across a broader range of thoracic–lumbar curvature combinations. Thus, identical sensor-derived continuous measurements may generate markedly different categorical representations when different numerical thresholds and classification rules are applied.

This finding is particularly relevant in paediatric populations because sagittal spinal alignment changes during childhood and pubertal growth [1,2,3]. Age has been associated with sagittal spinal alignment, whereas evidence for independent associations with weight status and general sports participation is less consistent [4]. Body composition has been related to postural characteristics in school-aged children [5,7], while a pilot study in preschool children found no statistically significant between-group differences in overall posture irregularities according to body weight status [6]. Classification therefore imposes discrete decision boundaries on continuous quantitative variables reflecting sagittal spinal curvature. Sagittal posture categories should consequently be regarded as operational representations of measured spinal alignment rather than as naturally discrete biological entities. Different categorical and morphotype-based approaches used in paediatric populations further illustrate that the description of sagittal posture depends partly on the adopted classification framework [20,21,22].

The cross-classification results demonstrate this dependence particularly clearly. Several thoracic–lumbar combinations distinguished by the nine-type framework were aggregated within broader categories of the five-category system. Most notably, all 247 participants whose thoracic kyphosis and lumbar lordosis were both within the reference intervals adopted in the nine-type typology were classified as having round back according to the operationalised five-category rules. This discrepancy does not indicate inconsistency in the DIERS measurements, because the same continuous values were used in both classifications. Rather, it results directly from differences in numerical thresholds and category definitions. Consequently, terms such as normal posture, round back, or increased kyphosis should not be assumed to represent quantitatively equivalent conditions across different classification systems. Differences in reported frequencies may reflect not only biological variation between populations but also differences in reference intervals, thresholds, and category architecture [20,21,22]. Studies using categorical posture outcomes should therefore report the exact decision rules applied and, whenever possible, retain the underlying continuous measurements. The nine-type Wilczyński typology [23] uses a two-dimensional 3 × 3 structure in which thoracic kyphosis and lumbar lordosis are each classified as reduced, within the adopted reference interval, or increased. Their combination generates nine mutually exclusive profiles and therefore retains greater categorical detail regarding specific thoracic–lumbar combinations than the broader five-category system. However, greater categorical granularity is a structural characteristic of the classification and should not be interpreted as evidence that the resulting profiles constitute distinct pathophysiological entities, compensatory mechanisms, or clinically superior categories. Global sagittal balance, pelvic morphology, neuromotor mechanisms, and longitudinal adaptation were not assessed in the present study.

Shannon entropy quantified differences in category-distribution structure [24]. Entropy was higher for the nine-type typology than for the five-category classification (*H* = 1.87 vs. 0.91). Because the theoretical maximum entropy increases with the number of available categories, the higher absolute entropy of the nine-category system was partly structurally expected. Normalised entropy was therefore also calculated and remained higher for the nine-type framework (0.85 vs. 0.57). Thus, the nine-type typology generated a more even distribution relative to its own theoretical maximum. Nevertheless, neither absolute nor normalised Shannon entropy measures diagnostic accuracy, clinical validity, measurement precision, prognostic value, or therapeutic relevance. Normalisation improves comparison relative to the theoretical maximum of each system but does not convert entropy into a criterion of clinical validity. The finding supported by the present data is therefore limited to greater distributional evenness and categorical dispersion under the predefined classification rules; it does not demonstrate clinical superiority.

The cross-classification statistics require similar caution. The complete 9 × 5 contingency table yielded χ^2^ = 1142.80 and Cramér’s *V* = 0.55. However, both systems were deterministic transformations of the same two continuous variables. Cramér’s *V* should therefore be interpreted as a descriptive measure of cross-classification association rather than as evidence of agreement between independent diagnostic methods or validation of either classification. Moreover, 24 of the 45 expected cell counts were below 5, 14 were below 1, and 28 possible category combinations were structurally impossible under the predefined rules. Consequently, the assumptions underlying the conventional asymptotic Pearson chi-square test were not satisfied. Although the fixed-margin Monte Carlo sensitivity analysis with 100,000 replicates yielded *p* < 0.001, the simulation did not reproduce the complete structural-zero pattern. Its result should therefore not be interpreted as evidence of an independent statistical association between the systems.

The sensor-based measurement context must also be distinguished from the subsequent classification process. Rasterstereography is a non-contact, radiation-free optical measurement method based on photogrammetric reconstruction of back-surface geometry, objective localisation of anatomical landmarks, and model-based estimation of spinal shape [8,9,10,11,12,13,25]. Its reliability and validity have been evaluated under different acquisition conditions and for different parameters [14,15,16,17,18,19]. Measurement performance is parameter- and protocol-specific, and surface-topography-derived parameters should not be regarded as directly interchangeable with radiographic measurements [15,17,19]. An important conceptual distinction therefore exists between measurement validity and classification validity. Measurement validity concerns whether the sensor-derived kyphosis and lordosis parameters are measured reliably and represent the intended geometrical characteristics. Classification validity concerns whether the categories subsequently generated from those continuous measurements correspond to independent clinical, functional, prognostic, therapeutic, or longitudinal outcomes. Evidence supporting the measurement properties of rasterstereography therefore does not automatically validate the categorical system subsequently applied to those measurements. Measurements located close to decision thresholds also require caution because small variations related to habitual postural fluctuation, positioning, respiration, surface reconstruction, or measurement uncertainty may alter category assignment. Although standardised positioning and the 4D Average acquisition mode were used to reduce transient variability, category-level stability was not directly evaluated.

### 4.1. Strengths and Limitations

A principal strength of the present study is the relatively large sample of 952 children aged 10–12 years. Both classification frameworks were applied to exactly the same individual sensor-derived measurements of thoracic kyphosis and lumbar lordosis, thereby eliminating variability attributable to different acquisition technologies or separate examinations and allowing the influence of classification structure itself to be examined directly. The use of quantitative, non-ionising, sensor-based three-dimensional surface topography is also advantageous in paediatric research. In addition, category assignment was deterministic and based on predefined rules, eliminating examiner judgement from the final allocation after the continuous measurements had been obtained. Both absolute and normalised Shannon entropy were used to characterise distributional structure.

Several limitations should be acknowledged. Participants constituted a non-probability convenience sample recruited from schools in one region of Poland. Complete recruitment records were unavailable; therefore, participation rates could not be calculated, and selection bias cannot be excluded. The observed frequencies should consequently not be regarded as nationally representative prevalence estimates. The cross-sectional design also prevents assessment of changes in sagittal profiles during growth. Biological maturation was not directly determined using Tanner stage, skeletal age, peak height velocity, maturity offset, or comparable measures. Sex-specific analyses were outside the predefined methodological objective. Future studies should examine whether classification behaviour varies according to sex and biological maturation and should consider other potential sources of interindividual variability, including weight status, sports participation, and body composition [4,5,6,7].

The analysis was restricted to thoracic kyphosis and lumbar lordosis. Other DIERS parameters, including pelvic position, frontal-plane deviation, surface rotation, and trunk alignment, were not incorporated. No standardised clinical or radiographic scoliosis-screening protocol was systematically applied to the complete cohort; therefore, previously unrecognised frontal-plane or rotational abnormalities cannot be entirely excluded. The archived acquisition records did not permit retrospective confirmation of the exact number of frames included in the original 4D Average measurements, which limits exact technical replication. Furthermore, repeated measurements specifically designed to evaluate category-level test–retest stability were not performed.

The five-category framework also requires qualification. Its numerical rules represent an *a priori* operationalisation of conventional sagittal posture concepts described in the literature [20,21,22], rather than a universally accepted and independently validated five-category diagnostic algorithm. The comparison therefore applies specifically to the operationalised framework used in the present study.

The present sample partially overlapped with the cohort used in the earlier study in which the nine-type Wilczyński typology was originally described [23]. Specifically, all 303 participants from the original developmental cohort were included in the present sample of 952 children, corresponding to 31.8% of the current dataset. Consequently, the present study does not constitute an independent external validation of the nine-type Wilczyński typology or its threshold values; rather, it evaluates the categorical distribution generated by the predefined framework in an enlarged dataset. Figure 1 serves solely as an author-created schematic illustration of the 3 × 3 classification structure and should not be interpreted as evidence supporting the validity or validation of the typology.

Most importantly, no independent clinical, functional, prognostic, therapeutic, or longitudinal reference outcome was included. The present study therefore cannot establish whether the additional categorical differentiation observed with the nine-type framework corresponds to meaningful differences in symptoms, function, balance, motor performance, progression, treatment response, prognosis, or longitudinal stability. The investigation evaluates classification behaviour, not classification validity.

### 4.2. Practical Implications and Future Research

The present findings support practical recommendations for sensor-based posture research. When continuous sensor-derived thoracic kyphosis and lumbar lordosis measurements are transformed into categorical profiles, the resulting distribution depends on the numerical thresholds, number of categories, and classification algorithm applied. Exact criteria should therefore be reported explicitly, and the original continuous sensor-derived measurements should be retained and reported alongside categorical results whenever feasible. Continuous values preserve information that may otherwise be lost during categorisation and allow subsequent reanalysis when alternative thresholds or classification frameworks are considered. This is particularly important in comparative and longitudinal studies because differences in classification rules, software-derived parameters, or post-processing procedures may generate apparent differences in posture-profile distributions without corresponding differences in the underlying sensor-derived measurements.

The present findings do not justify recommending the nine-type typology as a superior diagnostic, screening, monitoring, or treatment-planning system. Its 3 × 3 architecture may be useful in research when the objective is to distinguish specific combinations of thoracic and lumbar curvature, but whether this additional granularity is clinically meaningful requires independent external validation. Potential validation domains include motor and gait function [28], muscle-related characteristics [29], neurological populations [30], postural control and balance [31,32,33], and behavioural or ergonomic factors related to sagittal alignment [34,35].

Future studies should use independent paediatric cohorts that do not overlap with the original developmental sample, include different age groups and stages of biological maturation, and evaluate potential sex-specific differences. Repeated-measurement designs are needed to establish category-level reliability and quantify the risk of reclassification near decision thresholds. Most importantly, future studies should determine whether specific sagittal profiles correspond to independently measured clinical, functional, prognostic, or longitudinal outcomes. Such outcomes, rather than entropy or category number alone, should determine whether greater classification granularity provides clinically meaningful information.

The central implication of the present study is therefore not that one classification should replace another. Rather, categorical representations of sagittal posture should be interpreted in relation to the numerical thresholds and algorithms that generated them. Transparent reporting of sensor acquisition and measurement procedures, continuous parameters, classification boundaries, and post-processing rules is essential for reproducibility and meaningful comparison across sensor-based posture studies.

## 5. Conclusions

The present study demonstrated that the categorical representation of sagittal posture depends substantially on the thresholds and classification structure applied to sensor-derived continuous measurements of thoracic kyphosis and lumbar lordosis. The operationalised five-category classification concentrated 94.1% of participants in two categories, whereas the nine-type Wilczyński typology produced a more dispersed and more evenly distributed set of sagittal postural profiles, as reflected by higher normalised Shannon entropy (0.85 vs. 0.57). The two-dimensional 3 × 3 structure of the nine-type typology provides greater categorical granularity by distinguishing specific combinations of thoracic kyphosis and lumbar lordosis that may be aggregated within broader categories of the five-category framework. However, greater categorical granularity and higher entropy should not be interpreted as evidence of superior diagnostic validity, clinical utility, prognostic value, or therapeutic relevance. Moreover, because both classifications were deterministic transformations of the same sensor-derived continuous measurements and the present cohort partially overlapped with the sample used to develop the nine-type typology, the study should not be regarded as an independent external validation of either classification system. The findings demonstrate that different predefined thresholds and classification algorithms can generate substantially different categorical representations from the same underlying sensor-derived measurements. From a measurement-science perspective, sensor-derived continuous parameters and the categorical algorithms subsequently applied to them represent distinct analytical levels and should be reported separately to support reproducibility and comparability across sensor-based posture studies. Whether the greater granularity of the nine-type typology provides additional clinical, functional, prognostic, or longitudinal value requires independent validation in external paediatric populations using appropriate reference outcomes.

## Figures and Tables

**Table 1:** Characteristics of the study population (*N* = 952).

Variable	Value
Age (years), mean ± SD	10.91 ± 0.79
Girls, *n* (%)	478 (50.2)
Boys, *n* (%)	474 (49.8)
Thoracic kyphosis angle (°), mean ± SD	41.12 ± 11.53
Lumbar lordosis angle (°), mean ± SD	40.49 ± 10.12

Note: Values are presented as *n* (%) for categorical variables and as mean ± standard deviation (SD) for continuous variables.

**Table 2:** Distribution of Sagittal Postural Profiles According to the Operationalised Five-Category Classification.

Postural Type	*n*	%
Round back	510	53.6
Normal posture	386	40.5
Round-concave back	33	3.5
Flat back	21	2.2
Concave back	2	0.2
Total	952	100.0

**Table 3:** Distribution of Sagittal Postural Profiles According to the Nine-Type Wilczyński Typology.

Postural Type	*n*	(%)
Thoracic kyphosis within the adopted reference interval, lumbar lordosis within the adopted reference interval	247	25.9
Reduced thoracic kyphosis, lumbar lordosis within the adopted reference interval	241	25.3
Reduced thoracic kyphosis, reduced lumbar lordosis	146	15.3
Thoracic kyphosis within the adopted reference interval, increased lumbar lordosis	108	11.3
Reduced thoracic kyphosis, increased lumbar lordosis	66	6.9
Thoracic kyphosis within the adopted reference interval, reduced lumbar lordosis	60	6.3
Increased thoracic kyphosis, increased lumbar lordosis	57	6.0
Increased thoracic kyphosis, lumbar lordosis within the adopted reference interval	25	2.6
Increased thoracic kyphosis, reduced lumbar lordosis	2	0.2
Total	952	100.0

**Table 4:** Cross-Classification of Sagittal Postural Profiles between the Five-Category Classification and the Nine-Type Wilczyński Typology.

Postural Type	Round Back	Round-Concave Back	Flat Back	Concave Back	Normal Posture	Total
Reduced thoracic kyphosis, reduced lumbar lordosis	15	0	21	0	110	146
Reduced thoracic kyphosis, lumbar lordosis within the adopted reference interval	20	0	0	0	221	241
Reduced thoracic kyphosis, increased lumbar lordosis	9	0	0	2	55	66
Thoracic kyphosis within the adopted reference interval, reduced lumbar lordosis	60	0	0	0	0	60
Thoracic kyphosis within the adopted reference interval, lumbar lordosis within the adopted reference interval	247	0	0	0	0	247
Thoracic kyphosis within the adopted reference interval, increased lumbar lordosis	97	11	0	0	0	108
Increased thoracic kyphosis, reduced lumbar lordosis	2	0	0	0	0	2
Increased thoracic kyphosis, lumbar lordosis within the adopted reference interval	25	0	0	0	0	25
Increased thoracic kyphosis, increased lumbar lordosis	35	22	0	0	0	57
Total	510	33	21	2	386	952

**Table 5:** Descriptive Cross-Classification Statistics for the Five-Category Classification and the Nine-Type Wilczyński Typology.

Statistic	Value
Sample size (*N*)	952
χ^2^	1142.80
Nominal df	32
Cramér’s *V*	0.55
Expected cells < 5	24/45 (53.3%)
Expected cells < 1	14/45 (31.1%)
Minimum expected count	0.004
Monte Carlo *p*-value (100,000 replicates)	<0.001

**Table 6:** Shannon Entropy of Sagittal Postural Profile Distributions According to Classification System.

Classification System	Number of Categories (*k*)	Shannon Entropy (*H*)	Maximum Entropy (*Hmax*)	Normalised Shannon Entropy Index (*H*/*Hmax*)
Operationalised five-category classification	5	0.91	1.61	0.57
Nine-type Wilczyński typology	9	1.87	2.20	0.85

Note: *H*, Shannon entropy; *Hmax* = ln(*k*), maximum entropy for *k* categories; *H*/*Hmax*, normalised Shannon entropy index ranging from 0 to 1, with higher values indicating a more even distribution across the available categories.

## Data Availability

The datasets generated and/or analyzed during the current study are not publicly available due to privacy and ethical restrictions related to the participation of minors but are available from the corresponding author on reasonable request.

## Abbreviations

The following abbreviations are used in this manuscript:

3D	Three-dimensional
*H*	Shannon entropy
*Hmax*	Maximum Shannon entropy
*H*/*Hmax*	Normalised Shannon entropy index
ICT–ITL (max)	Thoracic kyphosis angle
ITL–ILS (max)	Lumbar lordosis angle
SD	Standard deviation
